# Psychometric assessment of the Persian translated version of the “medical artificial intlligence readiness scale for medical students”

**DOI:** 10.1371/journal.pone.0323543

**Published:** 2025-05-12

**Authors:** Nasrin Khajeali, Noushin Kohan, Sajjad Rezaei, Alia Saberi

**Affiliations:** 1 Educational Development Center, Ahvaz Jundishapur University of Medical Sciences, Ahvaz, Iran; 2 Department of Medical Education and Department of E-learning in Medical Education, Smart University of Medical Sciences, Tehran, Iran; 3 Department of Psychology, Faculty of Literature and Humanities, University of Guilan, Rasht, Iran; 4 Neurosciences Research Center, Poursina Hospital, Faculty of Medicine, Guilan University of Medical Sciences, Rasht, Iran; Sapporo Medical University, JAPAN

## Abstract

**Background:**

Artificial intelligence (AI) has recently entered the medical field, but the level of readiness of medical students for it is not obvious. A tool with appropriate psychometric properties for use in different languages and for international comparison is required to measure this readiness. Medical Artificial Intelligence Readiness Scale for medical students (MAIRS-MS) is most complete scale for this purpose till now.

**Objectives:**

The purpose was to evaluate the Psychometric properties of the Persian-translated version of the MAIRS-MS and verify the replication of the original factor structure in Persian.

**Materials and methods:**

This study was conducted at Guilan University of Medical Sciences in 2023. Validation of the Persian translated scale (P-MAIRS-MS) was performed by determining the face, content, and construct validity and reliability, impact Score, CVI, CVR, Cronbach’s alpha, McDonald’s omega, and ICC, and performing confirmatory factor analysis (CFA). AMOS26 and SPSS26 software were used.

**Results:**

The translated scale had good quantitative and qualitative face and content validity (all items had the Impact Score higher than 1.5, CVI >= 0.8 and CVR>= 0.8). CFA confirmed the appropriate fit of the four-factor model (χ^2^/df = 1.963, RMSEA-0.063, CFI = 0.939, GFI = 0.901). Convergent validity was suitable in the first- and second-order CFA (AVE > 0.5, CR > 0.7 CR > AVE for each factor except Ability). Cronbach’s alpha (α=0.938) and McDonald’s omega (ω= 0.938), and ICC (0.992) indicated acceptable reliability and reproducibility of the scale.

**Conclusion:**

The P-MAIRS-MS demonstrated good psychometric properties and can be used for measuring and international comparing the medical students’ readiness for AI.

## Introduction

Artificial intelligence (AI) is a new technology developed in recent years. It is used in various fields, especially medicine and health care. Some examples of its applications in these fields include computer programs for analyzing medical images, diagnostic systems in the form of apps, wearable devices and biosensors, and IoT (Internet of Things) devices that monitor people’s health and electrocardiogram and can take action if needed. AI can also enable remote surgery and virtual hospitals. This technology can benefit many medical fields, such as radiology, pathology, dermatology, surgery, ophthalmology, emergency medicine, epidemiology, and disease management [1–4].

AI is used in neurology to improve diagnosis and treatment tremors and other movement disorders and identify their types to enhance deep brain stimulation (one of the treatment approaches for tremors) results, differentiate true epilepsy from nonepileptic epilepsy, predicts outcomes of epilepsy surgery and detects patterns of sudden, unexpected death in epilepsy. Sophisticated neuroimaging algorithms classify cognitive disorders predict dementia prognosis. Smartwatches monitor atrial fibrillation to prevent ischemic stroke. [5–7].

In the pharmaceutical field, it plays a significant role in drug synthesis. Also, peptides are commonly used in the development of new chemical compounds and peptide designs [8].

AI systems in healthcare can help doctors save time by transcribing notes, entering patient data, and diagnosing patients. Patients can also use AI systems to learn about medications and treatments. AI can also be used to diagnose patients in rural areas, thereby improving healthcare service accessibility [9]. It can potentially improve healthcare by addressing issues such as medical errors, inaccurate diagnoses, and patient anxiety [10].

However, AI technology raises some questions and problems. Some researchers believe that it can replace doctors, whereas others believe that programming skills are required to use it effectively [1]. In some studies, medical students and physicians expressed positive opinions and awareness about the potential uses and benefits of AI in medicine. They believe that this can play a significant role in the medical community [5,9].

AI can impact society’s health beyond treatment and healthcare. Using AI in medical research and education can help improve health by applying high technology to enhance the medical education system. This can lead to a better understanding of lessons and promote evidence-based medicine. Therefore, it seems that now, incorporating AI training into medical education curricula for medical students is necessary.

Before implementing AI in health and medicine, its effectiveness and feasibility should be evaluated. Therefore, it is first recommended to study the cognitive and emotional readiness of the aimed population about it. To assess the problems of AI, understand the strengths and weaknesses of medical students in AI, international comparison, plan for incorporating AI training into the medical educational programs, and implement AI in each area of healthcare a valid and reliable scale is required in each country with its national language.

In this regard, different scales and questionnaires have been designed around the world, most of which investigate the attitude and knowledge of different medical communities, toward AI [9,11–18]. However, there is only one scale to assess the readiness of medical students for an artificial intelligence named “Medical Artificial Intelligence Readiness Scale for Medical Students” (MAIRS) which was designed by Karaca O. and colleagues in 2021 in the English language. It assesses four domains including cognition (knowledge), ability, vision (attitude), and ethics [11], and is more complete than the other scales or questionnaires which most of which investigate the attitude and knowledge. Also, the psychometric properties of the English version of this scale (MAIRS-MS) demonstrate that it is a valid and reliable tool for assessing the readiness of medical students regarding artificial intelligence technologies and applications [11].

Although it is newly (2021) designed instrument, it has been used in this regard in some researches in many countries and universities including in Malaysia [19,20], Saudi Arabia [21] and Jordan [22] and one study on the International Collaboration and Exchange Program (ICEP) [23].

To meet the need for a Persian instrument for assessing the level of readiness for medical AI and future planning and reaching the above-mentioned goals about AI among medical students in Iran, as the MAIRS-MS was the most perfect scale for this purpose till now, we chose it for translation and validation. Since we want to use it for international comparisons, it does not require a different factor structure. Therefore, the main objective of this study was to confirm the validity of the original MAIRS-MS factor structure within a Persian-language context by CFA, which verifies that the translated version preserves the theoretical framework of the original scale.

## Materials and methods

The study used a cross-sectional psychometric design.

Considering the ethics of the research, the proposal of this study was approved by the Ethics Committee of the Smart University of Medical Sciences, with the ethic code: IR.SMUMS.REC.1402.008. All stages of this study were conducted in adherence to the ethical guidelines outlined in the 2013 Declaration of Helsinki. Written permission was obtained from the main designer of the questionnaire (Karaca O. and colleagues) to translate. To collect data, the participants of the study were informed about the purpose and method of the study and assured that the collected information is confidential and the collected data will be published as the overall average of the group. Before collecting data and after considering the inclusion and exclusion criteria, informed consent was fulfilled by each medical student.

### Participants

The study included medical students of Guilan University of Medical Sciences (GUMS) in northern Iran, in 2023. The sample size was determined based on the requirements for factor analysis. It is advised to choose 5–10 subjects for each item [24,25]. For this study, we chose 10 individuals for each item. Since there are 22 items in the MAIRS-MS, and considering a 10% dropout rate, the sample size was determined as 245 medical students. The sampling method was quota sampling. We randomly selected participants from all educational levels, (including basic sciences, physiopathology, externs, and interns). The inclusion criteria were a willingness to take part in the study, Iranian nationality and Persian language. And exclusion criteria were the questionnaires which were answered by less than 80%.

### Data collection

To gather data, we used a checklist that included demographic and educational information such as age, gender, and educational level. Also, we used the MAIRS-MS questionnaire, which was designed as a 5-point Likert scale ranging from strongly disagree to strongly agree [11] and is explained as follows. The questionnaire which was answered to more than 80% were acceptable and we continued sampling till 245 acceptable questionnaires were fulfilled.

### The Instrument for assessment and data gathering

In 2021, Karaca O. and colleagues introduced a tool called the Medical Artificial Intelligence Readiness Scale for Medical Students (MAIRS-MS) [11]. This tool assesses the preparedness of medical students for artificial intelligence technologies and their applications in medicine. It consists of 22 items categorized into four domains: cognition, ability, vision, and ethics. The results of analyses, including Exploratory Factor Analysis (EFA) and Confirmatory Factor Analysis (CFA), demonstrated that this scale possesses construct validity. Overall, it is considered a valid and reliable tool for evaluating the readiness of medical students in the field of artificial intelligence [11].

### Translation process

Initially, we obtained permission from its developers (Karaca O. et al.) to translate and use the MAIRS-MS questionnaire. Forward-backward translation was conducted. Two independent translators proficient in English conducted a forward-backward translation of the original English version of the questionnaire. After the second stage of forward translation, two translators with medical degrees and knowledge of artificial intelligence independently translated the MAIRS-MS. Following this, the study team formed a panel to discuss and compare both translations, identifying any contradictions or differences. These were then corrected and combined into a single copy, resulting in the preparation of the Persian version of the MAIRS-MS questionnaire (P-MAIRS-MS).

Concerning back translation, the two translators were asked to cooperate with the research. They were also familiar with the translation of educational scales and questionnaires. Back translation review and harmonization were done to ensure conceptual equivalence. Finally, the final Persian version was translated into English and has been compared with the original version of the questionnaire, and it was eventually approved [26].

### Data analysis

The main objective of this study is not to discover a new factor structure, but rather to confirm the validity of the original MAIRS-MS factor structure within a Persian-language context, which verifies that the translated version preserves the theoretical framework of the original scale and remains comparable on an international level. Since EFA was already conducted during the development of the original MAIRS-MS, study by Karaca et al., 2021[11], and our goal is to ensure international comparability, therefore, Confirmatory Factor Analysis (CFA) alone is methodologically appropriate and sufficient for validating the translated version of MAIRS-MS in the Persian context. Conducting EFA could reveal a different factor structure, which would undermine our objective of maintaining consistency with the original scale, thereby preventing international comparisons, which contradicts the study’s objective.

In this study, validation of the Persian translated scale (P-MAIRS-MS) was performed by determining the face, content, and construct validity and reliability, impact Score, CVI, CVR, Cronbach’s alpha, McDonald’s omega, and ICC, and performing Confirmatory Factor Analysis (CFA). AMOS26 and SPSS26 software were used.

### Face validity

The face validity of the P-MAIRS-MS was measured by quantitative and qualitative methods. In the quantitative validity analysis, the opinions of 10 medical students were used (They were asked to express the importance of the scale’s items based on a 5-point Likert scale (from completely important to not important at all). Then, item impact, were calculated according to the following formula: Impact Score = Frequency (%) × Importance. Frequency is the number of people who gave 4 and 5 points to each item, and Importance is the average score obtained from the responses of the participants to the above-mentioned Likert scale. Only questions with a score higher than 1.5 were acceptable in terms of face validity.

The qualitative analysis of face validity is performed on the items which in the quantitative assessment of face validity had the determined impact score of less than 1.5. It involved face-to-face interviews with the target group to comment on the level of difficulty, relevance, and ambiguity in expressions of the phrases and inadequacy in the meanings of words in each of the items. Their comments will be applied in the form of minor changes in the questionnaire [27].

### Content validity

The content validity of the P-MAIRS-MS was assessed by quantitative and qualitative methods.

In the qualitative content validity evaluation phase, P-MAIRS-MS was given to 10 qualified experts who were familiar with the field of artificial intelligence in medicine. They were asked to provide their correct views in writing regarding each item after a detailed study of the tool. It was emphasized that compliance with the standards of grammar, use of appropriate words, placement of the items in their proper place, and appropriate scoring should be taken into consideration [28]. After collecting experts’ opinions, the necessary changes were made to the tool, based on the opinions provided. And 5 items were corrected in terms of grammar and use of appropriate words.

Quantitative content validity assessment was based on the opinion of the experts in that field, and it involved two indexes content validity ratio (CVR) and content validity index (CVI). In the CVR index, the necessity of an item was checked. The purpose of the content validity ratio is to select the most important and accurate content. The translated questionnaire was given to 10 medical professors who are familiar with artificial intelligence and its applications in medicine, and they were asked to examine the items in terms of necessity and score each item based on a 3-point scale (1. Not necessary; 2. Useful but not necessary; 3. Necessary). The minimum value of the CVR was determined based on Lawshe’s table (1975), according to the number of 10 people, 0.62. The following formula was used to calculate CVR: CVR =$\frac{nE\;-\;N/2}{N/2}\;$ [27].

Where nE is the number of experts who have considered the item in question as necessary, and N is the total number of experts.

CVI is calculated to ensure whether the items are designed in the best way to measure the constructs. For this purpose, Waltz & Bausell method was used. The tool was provided to another 10 qualified experts who were familiar with the field of artificial intelligence in medicine. They were asked to determine the degree of relevance of each item using a 4-point Likert scale for each of the items (from not relevant to completely relevant) [29]

CVI was calculated according to the following formula: CVI = Ne/N. Ne is the number of experts who have chosen options 3 and 4, and N is the total number of experts. If the score of an item is more than 0.79, that item is acceptable. If the score is between 0.70 and 0.79, the item needs to be revised, and if the score is less than 0.70, the item is unacceptable and should be removed [30].

### Construct validity

To determine the construct validity of the P-MAIRS-MS, confirmatory factor analysis (CFA) with the maximum likelihood ratio estimation method was used. The dataset of 245 eligible medical students of GUMS was used to conduct CFA. AMOS software version 24 was used to analyze data.

To check the absence of multivariate outlier data, the Mahalanobis distance index was examined and significance levels less than 0.05 indicate the remoteness of the desired outlier data. The assumption of univariate normality was checked based on the skewness index of ± 2 [31]. Mardia standardized kurtosis coefficient and critical ratio were used to check the multivariate normality. According to Blunch (2012) [32] if the critical ratio obtained from Mardia standardized kurtosis coefficient is less than 5, the assumption of multivariate normality has been met.

Jaccard and Wan suggested that at least six model fit indices should be presented to show that the model has a good fit [33]. In this study, some of the fit indices were used and calculated to assess the model fit. The included Chi-square (χ2) test, χ2/degree of freedom (df) ratio (CMIN/DF) [< 3 good, < 5 acceptable], Parsimonious Normed Fit Index (PNFI)>0.5, Parsimonious Comparative Fit Index (PCFI)> 0.5, Comparative Fit Index (CFI) >0.9, Incremental fit index (IFI) >0.9, Goodness of Fit Index (GFI) >0.9, Root Mean Square Error of Approximation (RMSEA) <0.08. [34]. the level of significance was set to 5%.

### Convergent validity

Convergent validity is the assessment to measure the level of correlation of multiple indicators of the same construct that are in agreement. To establish convergent validity of the P-MAIRS-MS structure in the first and second-order confirmatory factor analysis, the factor loading of the indicator, composite reliability (CR), and the average variance extracted (AVE) have to be considered. To establish convergent validity, the values of AVE > 0.5, CR > 0.7 and CR > AVE should be [35].

### Reliability

To determine the reliability index, internal consistency and stability of P-MAIRS-MS were measured. To evaluate the internal consistency of P-MAIRS-MS, Cronbach’s alpha (α), and McDonald Omega coefficient (ω), were estimated at values greater than 0.7, which indicates the internal consistency of the tool [18]. To determine the stability of P-MAIRS-MS, the test-retest method was used to determine the intraclass correlation coefficient (ICC) index, and 39 of the subjects who responded to P-MAIRS-MS in the first stage, completed the mentioned questionnaire after four weeks. To estimate the ICC, the absolute agreement method with the two-way random model was used. (ICC < 0.8: very favorable reliability, 0.8–0.6: moderate reliability, and > 0.6: poor reliability) [36].

## Results

This study investigated 245 medical students of GUMS with an average age of 22.12 ± 2.91 (19–47) years. 137 students (55.9%) were female and 108 students (44.1%) were male.

### Face validity

In the quantitative face validity assessment, the calculated Impact Score of each item was higher than 1.5, and face validity was acceptable. As said in the method section, the qualitative analysis of face validity is performed on the items which in the *quantitative* assessment of face validity had the determined impact score of less than 1.5. As the calculated Impact Score was higher than 1.5 for all item, therefore, the qualitative assessment of face validity for each item wasn’t need.

### Content validity

The qualitative content validity was also confirmed after the tool was modified by 10 qualified experts who made the necessary grammatical corrections. In quantitative content validity, the results showed that all items had acceptable CVI (>= 0.8) and CVR (>= 0.8) and therefore good content validity. Thus, none of the items were deleted or changed.

### Normality of data

In the assessment of the normality of data, no outlier data were identified. The Skewness index was in the mean of ± 2, and the Merdia kurtosis coefficient and critical ratio were 4.011 and 2.219 respectively. So, the normality of data is established.

### Construct validity

**Fig 1:**
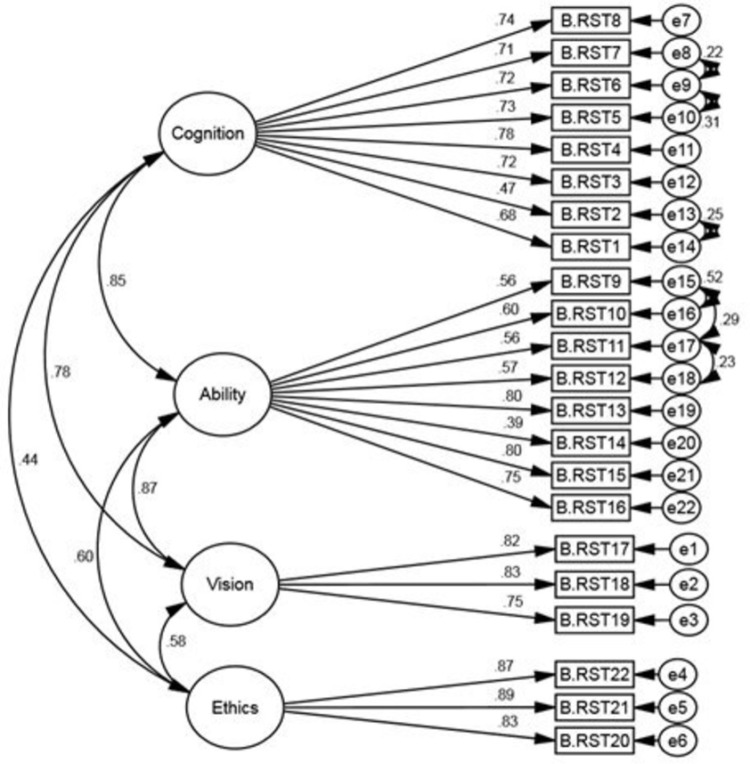
The structure of the P- MAIRS-MS; the modified model resulting from the 1^st^ order CFA (n = 245).

In the confirmatory factor analysis (CFA) at first, the measured goodness-of-fit indices showed a poor fit of the proposed model with the data. In the next step to improve the fitness of the proposed model, the correlation between the measured errors (e8-e9, e9-e10, e15-e16, e14-e13, e15-e17, and e17-e18) was drawn and the model fit indices were recalculated after modification. The indices of model fit before and after modification are shown in Table 1. After modifying the model, the chi-square index (χ2) was obtained as 382.909, (P < 0.001). Then, to evaluate the fit of the model, other indicators were also examined. All of the measured goodness-of-fit indices confirmed the appropriate fit of the model (Table 1). The standardized factor loadings of the revised four-factor CFA model of the P-MAIRS-MS construct are shown in Fig 1. The results show that the factor loading value of all the items of each factor is higher than 0.6 [35].

**Table 1:** The goodness of fit model indices of P- MAIRS-MS (1^st^ & - 2^nd^ order CFA).

*The goodness of fit model indices*	*χ* ^*2*^	*Df*	*P-value*	*CMIN/df*	*RMSEA (CL90%)*	*PNFI*	*CFI*	*PCFI*	*IFI*	*GFI*
Before modification	549.913	203	.001>	2.709	.084(.07-.09)	.732	.887	.779	.888	.813
After modification	382.909	197	.001>	1.944	.062(.05-.07)	.754	.939	.801	.940	.901
Second-order	390.556	199	<.001	1.963	.063(.05-.07)	.759	.938	.808	.938	.900

* PNFI, PCFI (>0.5), GFI,CFI, IFI(>0.9) RMSEA (<0.08), CMIN/DF (good<3, acceptable<5).

**Fig 2:**
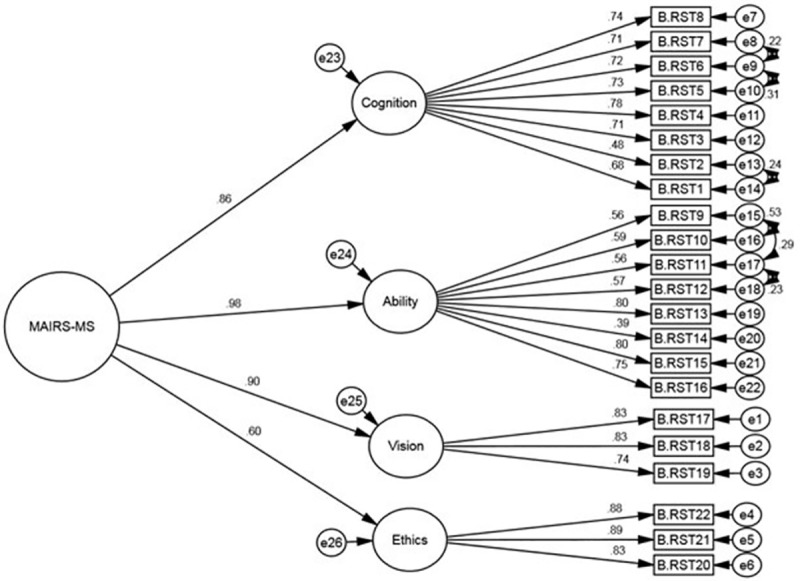
The structure of the P- MAIRS-MS; the modified model resulting from the 2^nd^ order CFA (n = 245).

After examining the correlation between the factors and identifying items in the first-order CFA, to determine the contribution of each factor including the Cognition, Ability, Vision, and Ethics factors in the explanation of P-MAIRS-MS structure, the second-order CFA method was used. Table 3 also shows the measured fit indices in the second-order CFA, which indicates the goodness of fit of the final four-factor model. Fig 2 shows the standardized factor loadings of each factor and construct in the second-order CFA.

**Table 2:** Convergent validity of P-MAIRS-MS structure.

*Factors*	*1*^*st*^ *order CFA*		*2*^*nd*^ *order CFA*	
	*AVE*	*CR*	*AVE*	*CR*
*Cognition*	0.50	0.88	0.72	0.91
*Ability*	0.42	0.84		
*Vision*	0.62	0.85		
*Ethics*	0.75	0.90		

*Abbreviations; CR: Construct Reliability; AVE: Average Variance Extracted.

**Table 3:** Results of internal consistency (n = 245) and instrument stability (Interclass Correlation Coefficient, n = 39) P-MAIRS-MS.

Factor	Cronbach’s alpha	McDonald Omega	ICC	CI (95%)		p-value
				*Lower band*	*Upper band*	
*P-MAIRS-MS*	.938	.938	.992	.961	.998	<.001
*Cognition*	.886	.889	.980	.843	.995	<.001
*Ability*	.851	.851	.964	.891	.988	<.001
*Vision*	.841	.844	.982	.937	.994	<.001
*Ethics*	.899	.900	.998	.994	.999	<.001

### Convergent validity

The results summarized in Table 2 demonstrate the convergent validity of the P-MAIRS-MS factors and the correlations between the items of each factor. For each factor except Ability, this condition is met: AVE > 0.5, CR > 0.7 CR > AVE.

### Reliability

The results summarized in Table 3 demonstrated the reliability of P-MAIRS-MS. Cronbach’s alpha and McDonald’s Omega coefficient and interclass correlation coefficient were in the acceptable range for each factor of P-MAIRS-MS.

## Discussion

Given the increasing demands and technological advancements in the medical field, it is important to assess the readiness for incorporating artificial intelligence in medicine. Several questionnaires and scales have been designed to investigate “knowledge and attitude” towards artificial intelligence in medicine, including “General Attitudes Towards Artificial Intelligence Scale” (GAAIS) designed by Schepman, and “Attitude towards Artificial Intelligence” in German, Chinese, and English languages, designed by Sindermann et al. These scales have been thoroughly validated and exhibits acceptable psychometric properties [12,14]. Additionally, some questionnaires have been developed, but they have not undergone psychometric evaluation and have only assessed the attitude and knowledge of medical students or physicians toward AI [8,9,13,15–18].

As previously stated, the only complete and comprehensive tool to measure medical students’ readiness toward AI was developed by Karaca et al. called “Medical artificial intelligence readiness scale for medical students” (MAIRS-MS). To evaluate psychometric properties of the original version of this scale, both Exploratory Factor Analysis (EFA) and Confirmatory Factor Analysis (CFA) were utilized. In Karaca et al study, there were 568 medical students in the EFA stage and 329 medical students in the CFA stage from two public universities in Turkey. The initial 27 items were narrowed down and finalized to 22 items in a four-factor structure (cognition, ability, vision, and ethics) [11]. The objective of the present study was to validate and standardize the Persian version of this scale and to verify the replication of the original factor structure in Persian for international comparison, rather than to discover new factors. Performing EFA for translated scale could alter the factor structure, thereby preventing international comparisons, which contradicts the study’s objective. Therefore, CFA alone is methodologically appropriate for validation in a translated version.

In the current study we found that the Persian version of MAIRS-MS (P- MAIRS-MS) had good psychometric properties as follows. Quantitative and qualitative face and content validity were acceptable. In the confirmatory factor analysis (CFA), the first and second-order goodness of fit indices confirmed the appropriate fit of the four-factor model. Convergent validity was also suitable in the first and second-order CFA. Cronbach’s alpha and McDonald’s omega values and ICC indicated acceptable reliability and reproducibility of the scale.

In assessing the qualitative face and content validity of the P-MAIRS-MS, the experts’ suggestions were considered and applied to the items if they were logical and accurate. The quantitative face and content validity were deemed acceptable based on the results. Compared to the original English version of the MAIRS-MS designed by Karaca et al., its quantitative and qualitative face and content validity of the P-MAIRS-MS were also accepted, with 27 items retained through exploratory factor analysis (EFA) [11].

To determine the construct validity, a confirmatory factor analysis (CFA) method was applied to a sample of 245 medical students from the Guilan University of Medical Sciences in Iran. Jaccard and Wan suggested that at least six model fit indices should be presented to show that the model has a good fit [33]. In this study, seven fit indices, including χ2, CMIN/DF, PNFI, PCFI, CFI, IFI, GFI, and RMSEA, were examined. The obtained indices from the modified initial model in both first and second-order factor analyses indicated a strong fit for the four-factor model of P-MAIRS-MS. Therefore, the Theoretical model resulting from the content validity study aligned with the Experimental model derived from the study on the target group. [34,36,37–39]

In the first-order confirmatory factor analysis of the modified four-factor model, all items of P-MAIRS-MS had a standardized factor loading value higher than 0.6, indicating a very positive relationship between each factor (hidden variable) and its sub-items (observable variables) [35]. The findings from the second-order factor analysis indicated that the factors of Cognition, Ability, Vision, and Ethics have a positive association with the main structure of P-MAIRS-MS and could be part of this structure. Additionally, the first-order CFA provided evidence for the convergent validity of the construct factors in P-MAIRS-MS, with only a slight weakness observed in the Ability factor. In the sense that all 4 factors (to a lesser extent: ability) have a high inter-item correlation. Also, the whole construct had good convergent validity in the second-order CFA, indicating a positive relationship between the whole construct and its factors. Also, in the CFA of the original English version of MAIRS-MS, 6 model fit indices were used and all were within the acceptable fitness interval [11].

To be ready to accept any new process in any structure, including medical sciences, it is necessary to have Cognition, Ability, Vision and belief, and compliance with Ethics. This principle applies to the integration of artificial intelligence into medical sciences and its utilization. MAIRS-MS included these four factors, and as we didn’t find any other comprehensive scale such as MAIRS-MS, therefore we compared the results of our study with the other studies that assess Cognition (Knowledge), Ability (behavior), Vision (insight and attitude) and Ethics in AI. In this regard, a tool named “Student Attitude toward Artificial Intelligence” (SATAI) was introduced to investigate the attitude of students towards artificial intelligence in Korea. SATAI evaluated students in three areas: behavioral, affective, and cognitive. This scale consisted of 26 questions and CFA confirmed its construct validity and goodness of fit model and convergent validity [40]. In a particular study, the authors created and validated a tool called “Attitudes toward Ethics of AI” (AT-EAI) to assess the attitudes of undergraduate students towards AI ethics. The latest modified version consists of 17 items and 5 dimensions, demonstrating acceptable reliability, content validity, and construct validity [41]. In the future, we can integrate these scales with MAIRS-MS to create a new, more comprehensive tool for evaluating the readiness of medical students for AI in medicine.

In determining the internal consistency (a part of reliability) of the P-MAIRS-MS, both Cronbach’s alpha and McDonald’s omega values exceeded 0.8 in both the overall scale and its subscales, indicating a high level of internal consistency for the P-MAIRS-MS. Notably, the highest alpha and omega values were associated with the *Ethics* factor. In the original English version of MAIRS-MS, Cronbach’s alpha coefficient was found to be above 0.7 for the overall scale as well as for the Cognition, Ability, and Vision factors, so the English version of MAIRS-MS has also high level of internal consistency in overall and also in these factors. However, it drops to 0.632 for the Ethics factor in English version of MAIRS-MS indicating it does not have favorable internal consistency [11]. Whether the original or Persian translated version of MAIRS-MS, demonstrate good internal consistency, but the internal consistency in Ethics factor was not the same and contradicts our findings which the highest alpha and omega values were obtained for the ethics factor. There could be several reasons for this discrepancy, such as cultural differences or variations in how research ethics are defined among different societies of the respondents. To measure the stability of the P-MAIRS-MS tool, 39 participants were asked to answer the questionnaires again after 4 weeks (test-retest method) and then the intraclass correlation coefficient was calculated. The test-retest method assumes that the variables or concepts to be measured as well as the characteristics of the examinees will not change during the time and the most acceptable test to determine the stability (reliability test) is the intraclass correlation coefficient. If the ICC value is higher than 0.8, the stability of the tool is at a very favorable level, if it is between 0.6 and 0.8, it indicates moderate reliability, and if it is less than 0.6, it indicates weak stability. In this study, the correlation coefficient of P-MAIRS-MS and its subscales was more than 0.9, which suggests a very favorable correlation between the two times of the test, and shows that P-MAIRS-MS has reliability and repeatability in the length of time. In the original English version of MAIRS-MS, correlations between the factors were also significant (p < 0.01) and the factors were related to each other [11].

Therefore, the results demonstrated the validity of the Persian-translated version of MAIRS-MS and highlighted its applicability in identifying strengths and weaknesses and medical students’ readiness to accept and use AI in practice. By searching for strengths (talent) and weaknesses, we can develop programs to strengthen talents and address the weaknesses of medical students in this area. Furthermore, having this kind of knowledge and also international comparison, enables us to perform more detailed planning in student training and empower students to apply AI. Furthermore, this data can be used in the strategic incorporation of AI training into medical educational programs and the effective implementation of AI across various domains within healthcare. However, suitable scales are required to detect these data, and the P-MAIRS-MS is the most comprehensive. Certainly, a valuable suggestion for future research is to consider merging scales that assess Cognition (Knowledge), Ability (Behavior), Vision (Insight and Attitude), and Ethics in the context of AI. This integration, particularly with MAIRS-MS, could pave the way for the development of more robust and comprehensive assessment tools. Additionally, translating these scales into various languages and subjecting them to rigorous psychometric testing in diverse language contexts will contribute to the refinement and validation of these instruments. Such endeavors hold the potential to design more perfected and universally applicable scales for the nuanced evaluation of Cognition, Ability, Vision or Attitude, and Ethics in or about AI or readiness for using AI in medicine.

### Limitations of the research

There was a possibility of answering the questions with low accuracy, but by describing the plan and the goals of the study, the students considered themselves obliged to respond with high accuracy.

## Conclusion

Overall, the results showed that the Persian translated MAIRS-MS had very good psychometric properties in a population of medical students and can be used as a tool to measure medical students’ readiness for AI in medicine.

In future studies, this scale can be used to assess the readiness of medical students at different medical universities and also among students in other paramedicine fields in Persian language countries and compare the results with the other countries.

Considering EFA could potentially alter the factor structure within the context of the other language, which would hinder international comparisons, however, this could serve as a novel topic for future research

## Supporting information

S1 File Gmail of permission for translation of MAIRS-MS from the designer of scale (Karaca O. et al, 2021). (PDF)

S2 File Rightslink by Copyright Clearance Center. (PDF)

S3 File MAIRS-MS (Medical Artificial Intelligence Readiness Scale for Medical Students). (PDF)

S4 File P-MAIRS-MS (Persian translation of MAIRS-MS). (PDF)
