# Supplementary material for: Psychometric assessment of the Persian translated version of the “medical artificial intlligence readiness scale for medical students”
Source: PLoS One. 2025 May 12;20(5):e0323543. doi: 10.1371/journal.pone.0323543 (PMC12068652; doi:10.1371/journal.pone.0323543)
Supplement: S2 File — (PDF) [file pone.0323543.s002.pdf]

RightsLink

Medical artificial intelligence readiness scale for medical students (MAIRS-MS) – development, validity and reliability study

Author: Ozan Karaca et al

Publication: BMC Medical Education

Publisher: Springer Nature

Date: Feb 18, 2021

Copyright © 2021, The Author(s)

Creative Commons

This is an open access article distributed under the terms of the Creative Commons CC BY license, which permits unrestricted use, distribution, and reproduction in any medium, provided the original work is properly cited.

You are not required to obtain permission to reuse this article.

CC0 applies for supplementary material related to this article and attribution is not required.

© 2025 Copyright - All Rights Reserved | Copyright Clearance Center, Inc. | Privacy statement | Data Security and Privacy | For California Residents | Terms and ConditionsComments? We would like to hear from you. E-mail us at customercare@copyright.com
