# Supplementary material for: Psychometric assessment of the Persian translated version of the “medical artificial intlligence readiness scale for medical students”
Source: PLoS One. 2025 May 12;20(5):e0323543. doi: 10.1371/journal.pone.0323543 (PMC12068652; doi:10.1371/journal.pone.0323543)
Supplement: S3 File — (PDF) [file pone.0323543.s003.pdf]

Medical Artificial Intelligence Readiness Scale for Medical Students (MAIRS-MS)

1-Strongly Disagree	2-Disagree	3-Neutral	4-Agree	5-Strongly Agree

1. I can define the basic concepts of data science
2. I can define the basic concepts of statistics
3. I can explain how AI systems are trained
4. I can define the basic concepts and terminology of AI
5. I can properly analyze the data obtained by AI in healthcare.
6. I can differentiate between the functions and features of AI related tools and applications.
7. I can organize workflows in accordance with the logic of AI.
8. I can express the importance of data collection, analysis, evaluation and safety; for the development of AI in healthcare.
9. I can use AI-based information in combination with my professional knowledge.
10. I can use AI technologies effectively and efficiently in healthcare delivery.
11. I can use artificial intelligence applications in accordance with its purpose.
12. I can access, evaluate, use, share and create new knowledge using information and communication technologies.
13. I can explain how AI applications in healthcare offer a solution to which problem.
14. I find it valuable to use AI for education, service and research purposes.
15. I can explain the AI applications used in healthcare services to the patient.
16. I can choose the proper AI application for the problem encountered in healthcare.
17. I can explain the limitations of AI technology.
18. I can explain the strengths and weaknesses of AI technology.
19. I can foresee the opportunities and threats that AI technology can create.
20. I can use health data in accordance with legal and ethical norms.
21. I can act in accordance with ethical principles while using AI technologies.
22. I can follow the legal regulations regarding the use of AI technologies in healthcare.

Cognition Factor: 1-8 Items Min:8 Max:40 points

Ability Factor: 9-16 Items Min:8 Max:40 points

Vision Factor: 17-19 Items Min:3 Max:15 points

Ethics Factor: 20-22 Items Min:3 Max:15 points

Medical Artificial Intelligence Readiness: 1-22 Items Min:22 Max:110 points

* Karaca, O., Çalışkan, S.A. & Demir, K. Medical artificial intelligence readiness scale for medical students (MAIRS-MS) – development, validity and reliability study. BMC Med Educ 21, 112 (2021). https://doi.org/10.1186/s12909-021-02546-6

The scale is licensed under a Creative Commons Attribution 4.0 International License (http://creativecommons.org/licenses/by/4.0/).
