# Supplementary material for: Psychometric assessment of the Persian translated version of the “medical artificial intlligence readiness scale for medical students”
Source: PLoS One. 2025 May 12;20(5):e0323543. doi: 10.1371/journal.pone.0323543 (PMC12068652; doi:10.1371/journal.pone.0323543)
Supplement: S4 File — (PDF) [file pone.0323543.s004.pdf]

Medical Artificial Intelligence Readiness Scale for Medical Students (MAIRS-MS)

پرسشنامه "مقیاس آمادگی هوش مصنوعی پزشکی برای دانشجویان پزشکی"

1- من می توانم مفاهیم اساسی "علم داده" را تعریف کنم.
2- من می توانم مفاهیم اولیه "آمار" را تعریف کنم
3- من می توانم توضیح دهم که چگونه سیستم های هوش مصنوعی ، آموزش داده می شوند.
4- من می توانم مفاهیم اولیه و اصطلاحات هوش مصنوعی را تعریف کنم.
5- من می توانم اطلاعات مربوط به مراقبت های بهداشتی-درمانی را که توسط هوش مصنوعی به دست آمده ، به طور مناسب تجزیه و تحلیل کنم.
6- من می توانم عملکرد و ویژگی های ابزارها و برنامه های " متفاوت" هوش مصنوعی را از هم افتراق دهم.
7- من می توانم "تغییرات سیستم کاری" را سازگار با هوش مصنوعی سازماندهی کنم.
8- من می توانم اهمیت جمع آوری ، تجزیه و تحلیل، ارزیابی و امنیت داده ها را برای گسترش استفاده از هوش مصنوعی در مراقبت های بهداشتی-درمانی بیان کنم.
9- من می توانم از اطلاعات هوش مصنوعی درکنار دانش حرفه ای خودم استفاده کنم.
10- من می توانم از فناوری های هوش مصنوعی به طور موثر و کارآمد در ارائه مراقبت های بهداشتی استفاده کنم.
11- من می توانم از برنامه (اپلیکیشن) های هوش مصنوعی مطابق با هدف آن استفاده کنم.
12- من می توانم با استفاده از فناوری های اطلاعات و ارتباطات به دانش جدید دسترسی داشته باشم، آن را ارزیابی و استفاده کنم، وبه اشتراک بگذارم و تولید علم کنم .
13- من می توانم توضیح دهم که چگونه برنامه های هوش مصنوعی برای هر مشکل در مراقبت های بهداشتی راه حلی ارائه می دهند.
14- من استفاده از هوش مصنوعی را برای اهداف آموزشی، خدماتی (بهداشتی-درمانی) و تحقیقاتی ارزشمند می دانم.
15- من می توانم برنامه های کاربردی هوش مصنوعی مورد استفاده در خدمات بهداشتی-درمانی را برای بیمار توضیح دهم.
16- من می توانم یک برنامه هوش مصنوعی مناسب ، برای مشکل بهداشتی -درمانی پیش آمده انتخاب کنم.
17- من می توانم محدودیت های فناوری هوش مصنوعی را توضیح دهم.
18- من می توانم نقاط قدرت و ضعف هوش مصنوعی را توضیح دهم.
19- من می توانم فرصت ها و تهدیدهایی را که فناوری هوش مصنوعی می تواند ایجاد کند، پیش بینی کنم.
20- من می توانم از داده های بهداشتی، مطابق با موازین قانونی و اخلاق استفاده کنم.
21- من می توانم دراستفاده از فناوری های هوش مصنوعی ، اصول اخلاق را رعایت کنم.
22- من می توانم از مقررات قانونی در مورد استفاده از فناوری های هوش مصنوعی در مراقبت های بهداشتی -درمانی پیروی کنم.

عامل شناختی: موارد ۱-۸ ،حداقل: ۸ حداکثر: ۴۰ امتیاز

عامل توانايي: موارد ۹-۱۶، حداقل: ۸ حداکثر: ۴۰ امتیاز

عامل دیدگاه: موارد ۷-۱۹ ، حداقل:۳ ، حداکثر: ۱۵ امتیاز

عامل اخلاق: موارد ۲۰-۲۲، حداقل: ۳ حداکثر: ۱۵ امتیاز

آمادگی هوش مصنوعی پزشکی: موارد ۱-۲۲، حداقل: ۲۲حداکثر: ۱۱۰ امتیاز

This scale was developed based on the scale of the study by Karaca O, Çalışkan SA, Demir K. Medical artificial intelligence readiness scale for medical students (MAIRS-MS)–development, validity and reliability study. BMC medical education. 2021 Dec;21:1-9. https://doi.org/10.1186/s12909-021-02546-6. Translation and validation have been made to the original scale. The orginal scale is licensed under a Creative Commons Attribution 4.0 International License (http://creativecommons.org/licenses/by/4.0/). The translation and validation of its translated form of MAIRS-MS were performed after the permission of its first developers.
