# Supplementary material for: Psychometric assessment of the Persian translated version of the “medical artificial intlligence readiness scale for medical students”
Source: PLoS One. 2025 May 12;20(5):e0323543. doi: 10.1371/journal.pone.0323543 (PMC12068652; doi:10.1371/journal.pone.0323543)
Supplement: S1 File — (PDF) [file pone.0323543.s001.pdf]

Gmail

Alia Saberi <alia.saberi1@gmail.com>

Permission for translation of MAIRS-MS

9 messages

Alia Saberi <alia.saberi1@gmail.com> Sun, Aug 7, 2022 at 1:59 PM
To: ayhan.caliskan@ege.edu.tr

Dear Ayhan Çalışkan

I am studying in Tehran Virtual University of Medical sciences (VUMS) in Medical Education course and me and my professors (professor Khajehali and professor Kohan) have decided to assess the psychometric properties of "persian translation" of Medical artificial intelligence readiness scale for medical students **(MAIRS-MS)** (according to the article:https://doi.org/10.1186/s12909-021-02546-6).

We need your permission for translation of this scale to Persian and assess the psychometric properties of its Persian version .

Thanks a lot and with best regards

Alia Saberi (MD)

Editor in Chief of Caspian Journal of Neurological Sciences

Neurology Department, Guilan University of Medical Sciences, Rasht, Iran.

S. Ayhan CALISKAN <ayhanca@gmail.com> Tue, Aug 9, 2022 at 10:27 PM
To: alia.saberi1@gmail.com
Cc: ozan karaca <ozankaraca10@gmail.com>, kademir07 <kademir07@gmail.com>

Dear Dr Alia Saberi,
Greetings from Turkey!
I hope this finds you well and in good health.
Please accept my sincere apologies for my late response.

It is great to receive your email and thank you very much for your interest in our research.
We are glad you are planning a research project using our developed scale.

With pleasure, we are giving our formal permission to use our scale titled "**Medical artificial intelligence readiness scale for medical students (MAIRS-MS)**" under the conditions enclosed below:
- Please include a statement of attribution and copyright on all copies of the instrument and/or any instrument developed from it.
- We request a pdf copy or a web link of your completed scientific research outcome (report, article etc.) that we can reach and download.

- We are giving this permission to you personally and request that you will use MAIRS-MS -*and/or any developed scale from it*- only for your research study and will not sell or use them with any compensation.

I am sending our MAIRS-MS questionnaire's English version attached to this email.
I must remind you that we have developed this scale in Turkish. After the development, validity and reliability study in Turkish, we translated our MAIRS-MS scale from Turkish to English and one independent academic reviewed, corrected and approved the final version of the attached English version of MAIRS-MS.
Although the English language adaptation has not yet been done, we believe that the English version would give similar validity and reliability results.
Please use the MAIRS-MS scale document which is attached to this email for your research.

If you need any further information, we will be happy to share it with you.

We wish you the best of luck with your studies.

Warm regards

S. Ayhan ÇALIŞKAN MD, PhD
Ozan KARACA MSc, PhD
Kadir DEMİR MSc, PhD

Artificial Intelligence in Medical Education Research Group
https://medicaleducationai.wordpress.com/
https://twitter.com/AiMeded
https://www.linkedin.com/company/ai-meded

S. Ayhan CALISKAN, MD, PhD
Associate Prof. of Medical Education
Ege University Faculty of Medicine
Department of Medical Education
T: +90-232-390 1832 F: +90-232-343 6770
M:+90-533-390 8989 E: ayhanca@gmail.com

Kimden: "Alia Saberi" <alia.saberi1@gmail.com>
Kime: "ayhan caliskan" <ayhan.caliskan@ege.edu.tr>

Gönderilenler: 7 Ağustos Pazar 2022 12:29:36
Konu: Permission for translation of MAIRS-MS

[Quoted text hidden]

EGE ÜNİVERSİTESİ

Bu elektronik posta ve onunla iletilen bütün dosyalar sadece göndericisi tarafindan almasi amaclanan yetkili gercek ya da tüzel kisinin kullanimi icindir. Eger söz konusu yetkili alici degilseniz bu elektronik postanin icerigini aciklamaniz, kopyalamaniz, yönlendirmeniz ve kullanmaniz kesinlikle yasaktir ve bu elektronik postayi derhal silmeniz gerekmektedir.

EGE ÜNİVERSİTESİ bu mesajin icerdigi bilgilerin doğruluğu veya eksiksiz oldugu konusunda herhangi bir garanti vermemektedir. Bu nedenle bu bilgilerin ne sekilde olursa olsun iceriginden, iletilmesinden, alinmasindan ve saklanmasindan sorumlu degildir.
Bu mesajdaki görüsler yalnizca gönderen kisiye aittir ve EGE ÜNİVERSİTESİ'nin görüslerini yansitmayabilir.

This e-mail and any attachments may contain confidential and privileged information. If you are not the intended recipient, please notify the sender immediately by return e-mail, delete this e-mail and destroy any copies. Any dissemination or use of this information by a person other than the intended recipient is unauthorized and may be illegal.

EGE UNIVERSITY makes no warranty as to the accuracy or completeness of any information contained in this message and hereby excludes any liability of any kind for the information contained therein or for the information transmission, reception, storage or use of such in any way whatsoever. The opinions expressed in this message belong to sender alone and may not necessarily reflect the opinions of EGE UNIVERSITY.

Medical Artificial Intelligence Scale (MAIRS-MS).docx
22K

Alia Saberi <alia.saberi1@gmail.com> Tue, Aug 9, 2022 at 10:53 PM
To: "S. Ayhan CALISKAN" <ayhanca@gmail.com>
Cc: ozan karaca <ozankaraca10@gmail.com>, kademir07 <kademir07@gmail.com>

Dear S. Ayhan ÇALIŞKAN, Ozan KARACA ,Kadir DEMİR
Thanks a lot for your response and also your permission,
We will consider and do all of your command

With best regards

Alia Saberi (MD)

Editor in Chief of Caspian Journal of Neurological Sciences

Neurology Department, Guilan University of Medical Sciences, Rasht, Iran.

[Quoted text hidden]
